# Community College Gerontology Programs: Revisiting and Re-Visioning Workforce, Career Pathways, and Transfer

**DOI:** 10.1093/geroni/igab046.558

**Published:** 2021-12-17

**Authors:** Jennifer (Jenny) Sasser, Roger Anunsen, Michael Faber

## Abstract

The session will focus on new and existing innovative ways that Community Colleges are effectively addressing workforce needs resulting from a rapidly aging population; the ever-expanding career pathways available to students in the field of gerontology; as well as the continuing higher education needs of students transferring to bachelor’s and graduate-level gerontology programs. A panel of Community College and University gerontology professionals, representing both the GSA Community College and Aging Workforce Interest Groups, will share the innovative ways that they are working to address the three focus areas of this symposium. We will also include opportunities for discussion with participants about their experiences with and ideas for addressing these issues.

